# Effects of Monochromatic Light on Growth and Quality of *Pistacia vera* L.

**DOI:** 10.3390/plants12071546

**Published:** 2023-04-03

**Authors:** Dhekra Abdouli, Sihem Soufi, Taoufik Bettaieb, Stefaan P. O. Werbrouck

**Affiliations:** 1 Laboratory for Applied In Vitro Plant Biotechnology, Ghent University, Valentin Vaerwyckweg 1, 9000 Ghent, Belgium; 2 Laboratory of Horticultural Sciences, National Agronomic Institute of Tunisia, University of Carthage, 43 Av. Charles Nicolle, Tunis 1082, Tunisia

**Keywords:** hyperhydricity, LED, shoot tip necrosis, pistachio, proliferation

## Abstract

Light-emitting diodes (LEDs) are popular as a light source for in vitro plants because they save energy and allow the morphology of the plant to be altered. The purpose of this study was to show that switching from classical fluorescent light (FL) to LED light can have both beneficial and adverse effects. *Pistacia vera* plantlets were exposed to FL, monochromatic Blue LED light (B), monochromatic Red LED light (R), and a 1:1 mixture of both B and R (BR). R increased the total weight, shoot length, number of shoots ≥ 1 cm, and proliferation. It also reduced hyperhydricity (HH), but also dramatically increased shoot tip necrosis (STN) and leaf necrosis (LN). B cured plants of HH and STN, but hardly enabled proliferation. It did not solve the problem of LN, but the plants were high in total chlorophyll and carotenoids. BR reduced HH but enabled limited proliferation, high STN, and LN. All three LED treatments reduced HH compared to FL. B induced both high total phenolic and flavonoid content and high DPPH-scavenging activity. These results show that switching from FL to LED can have a significant positive or negative effect on proliferation and quality. This suggests that finding an optimal lighting regimen will take a lot of trial and error.

## 1. Introduction

*Pistacia vera*, member of the Anacardiaceae family and commonly known as pistachio, is native to the arid areas of central and western Asia and is widespread throughout the Mediterranean basin [1]. In Tunisia, the main producing country in the southern part of the Mediterranean basin, the pistachio tree has been propagated via grafting onto *Pistacia vera* seedlings [2]. However, climate change and monoculture are now threatening the sustainability of this crop [3]. The durability of this crop therefore requires the selection of adapted, tolerant, and efficient plant material. 

Plant tissue culture allows the production of identical plant clones with desired characteristics. Nowadays, there is a high demand for in vitro cloned pistachio rootstocks, such as selected drought- and salt-tolerant *Pistacia vera* clones. It is therefore unfortunate that their micropropagation still has some bottlenecks, such as hyperhydricity (HH), shoot tip necrosis (STN), and leaf necrosis (LN) [4,5]. Attempts to mitigate these physiological disorders by adjusting medium composition, gelling agent, subculture duration, and cytokinin type and concentration have not led to a comprehensive solution thus far [4,5,6,7]. However, plant tissue cultures are not only affected by the above factors, but are entirely dependent upon artificial light sources for illumination [8]. It is surprising that, to date and to the best of our knowledge, no research has been carried out on the effect of the light spectrum on the morphology and physiology of *Pistacia vera*. 

In heterotrophic in vitro plants, light is not considered a major source of energy. However, it is necessary as a crucial signal for plant growth and development, since it regulates various molecular, biochemical, and morphological processes [9,10]. Both primary and secondary metabolisms, including phenolic compounds, flavonoids, and other chemicals, are affected by light. The lighting sources that are most frequently used for in vitro culture are tubular fluorescent lamps that emit a wide spectrum, ranging from 350 to 750 nm, which contains wavelengths that are not utilized by plant culture.

Recently, tissue culture laboratories have switched to light-emitting diodes (LEDs) as a cost-effective alternative light source for optimizing plant growth conditions. LEDs are a fascinating tool, and by adjusting the Red/Blue ratio, intensity, and photoperiod, plant morphology (shoot length, leaf size, and branching) can be fine-tuned [11,12]. Red and Far-Red light can be used to manipulate phytochrome-dependent responses, and the same can be done with cryptochrome-dependent reactions that depend on Blue light [13,14]. In many previous studies, the regulatory effects of LEDs on the growth and development of plants such as peanut [15], grape [16], *Ficus benjamina* [17] and *Lippia alba* [18] have been observed.

Apart from the regulatory effects, manipulation of the spectral quality of LEDs significantly mitigated some physiological disorders, such as HH in *Lippia grata* culture [19].

In this study, we exposed *Pistacia vera* shoots to different light treatments and investigated whether LEDs could alleviate the associated physiological disorders without affecting the proliferation rate. We also examined the impact of LEDs on photosynthetic pigment production and antioxidant activity.

## 2. Materials and Methods

### 2.1. Stock Culture

Defoliated nodal segments (1 cm) of *Pistacia vera* were subcultured in 380 mL of Pistachio Optimal Medium (POM) [6] supplemented with 3% sucrose and 0.7% Plant Agar, to which 10 μM *mT* was added before adjusting pH to 5.7 and autoclaving for 20 min at 121 °C [5]. The cultures were maintained at 25 °C under a 16 h photoperiod. They were subcultured every six weeks for at least three years. 

### 2.2. Light Experiment

Five defoliated nodal explants were inoculated into 380 mL glass vessels that contained 100 mL of POM and had a cotton filter in the lid. A total of 45 explants were used per treatment. Four light treatments were used: the control treatment consisted of cool fluorescent light (FL) supplied by PHILIPS master TLD 36 W 830 Reflex ECO (40 μmol m^−2^ s^−1^ PAR) was compared with 100% Blue LED (B) (454 nm), 100% Red LED (R) (660 nm) and 50% B + 50% R (BR). The total photosynthetic photon flux density (PPFD) was set at 40 μmol m^−2^ s^−1^. Separate boxes (57 cm × 40 cm × 37.5 cm) with white walls and doors were built on the culture racks, mounted with Philips Green Power LED Research Modules, as described by [20]. After six weeks of culture, the effect of the light treatments was evaluated.

### 2.3. Morphological Parameters

Each shoot was separated into shoot and callus sections, which were weighed. The total and relative weight of callus and shoots was calculated. The total number of shoots per explant was counted, as well as the average number of “usable shoots” (≥1 cm) per explant. For each usable shoot, the length, total number of leaves, percentage of necrotic leaves, and percentage of shoots with shoot tip necrosis were recorded. The proliferation rate was estimated as the sum of the rounded length of each usable shoot (in cm) per explant, assuming that each internode was 1 cm long [5].

### 2.4. Determination of Percentage of Apoplastic Water and Hyperhydricity

The volume of apoplastic water was measured according to Kemat et al. [21]. The leaves were detached and weighed, and then transferred into a 2 mL collection tube, in which a QIAshredder spin column (DNeasy^®^ Plant Mini Kit—QIAGEN) was placed. The leaves were centrifuged at 3000 *g* for 20 min at 4 °C and were weighed again immediately afterward. The volume of apoplastic water (V_water_) in mL g^−1^ FW was calculated according to the formula of [22]: V_water_ = (FW − W_ac_)/FW × ρH_2_O, where FW = fresh leaf weight in mg, W_ac_ = fresh leaf weight after centrifugation, and ρH_2_O = water density (the water density was set equal to 1 g mL^−1^).

The apoplastic air volume of the leaves was determined with a pycnometer [21]. The leaves were cut, weighed, and then placed in the pycnometer, which was filled with distilled water and sealed with a stopper. The weight of the entire pycnometer, including the leaves, was measured, after which the stopper was removed and replaced with a plug of cotton. The pycnometer was placed under vacuum for five minutes to remove the intracellular air from the leaves. This was repeated until all of the air in the apoplast was replaced with water, causing the leaves to sink to the bottom. The pycnometer was completely filled with water and sealed with a glass stopper. After the outside was carefully dried, the weight of the pycnometer was determined. The apoplastic air volume (V_air_) in mL g^−1^ FW was calculated using the formula of Van den Dries et al. [22]: V_air_ = (W_av_ − W_bv_)/FW × ρH_2_O, where W_bv_ = weight in mg of the pycnometer including leaves and water before vacuum filtration, W_av_ = weight of the pycnometer including leaves and water after vacuum filtration, FW = fresh weight of the leaves, and ρH_2_O = water density. The percentage of water in the apoplast was calculated using the following formula [21]: %V_water_ = 100 × V_water_/(V_air_ + V_water_). The percentage of hyperhydric shoots was also visually estimated and calculated as (number of hyperhydric shoots per explant∗100)/(total usable shoots per explant). 

### 2.5. Pigment Content

The determination of chlorophyll a, b, and total was performed according to the methods of Arnon [23]. Total carotenoids were determined according to Lichtenthaler [24]. Chlorophyll extraction was performed using a mixture of acetone and water at a ratio of 80–20% (*v*/*v*). Fifty mg of dried leaves were homogenized with 5 mL of 80% acetone solution using a mortar and pestle. The filtrate was kept in the dark to prevent the oxidation of chlorophyll by light. Absorbance was measured at 663 and 645 nm. Chlorophyll concentration (a, b, and total) and carotenoids were expressed as µg/mL of acetone extract. 

### 2.6. Total Phenolic Compound (TPC)

TPC was determined by the Folin–Ciocalteu (F–C) method according to the description of [25]. Fifty mg of dry leaves from each sample were homogenized with 5 mL of ice-cold 95% (*v*/*v*) methanol. The samples were incubated for 48 h in the dark at room temperature and then centrifuged (13,000 *g* for 5 min at room temperature) and the supernatants were collected. One hundred µL of each sample’s supernatant in standard or 95% (*v*/*v*) methanol was added in duplicate to 2 mL microtubes. Subsequently, 200 µL of 10% (*v*/*v*) (F–C) was added and vortexed. Thereafter, 800 µL 700 mM Na_2_CO_3_ was added to each tube and incubated for 2 h at room temperature. Finally, absorbance was read at 765 nm. TPC was expressed as mg gallic acid equivalents per ml of methanol extract.

### 2.7. 1,1-Diphenyl-2 Picrylhydrazyl (DPPH) Scavenging Activity

DPPH was performed according to [26]. The methanol extraction was the same as that previously used for the determination of TFC. Methanol dilution was performed in order to bring the absorbance of the extract to a wavelength of 517 nm to 1.1 ± 0.02. After performing the previously described steps, 0.5 mL of sample was added to a test tube. The control sample was replaced with methanol. Subsequently, 1.5 mL of 0.3 mM DPPH solution (OD nm = 1.1 ± 0.02) was added to the test tube and incubated in the dark for 30 min. The DPPH-scavenging activity was determined according to the following formula:

DPPH-scavenging activity (%) = (Abs control − Abs sample) × 100/Abs control(1)

### 2.8. Determination of Total Flavonoid Content (TFC)

The TFC was measured by the aluminum chloride colorimetric method [27]. Briefly, 0.5 mL of previously used methanol extract or standard solution was mixed with 0.1 mL of 10% AlCl_3_. Thereafter, 1 mL 1 M CH_3_COOK and 4.3 mL distilled water were added, and the mixture was shaken vigorously. Quercetin was used as a standard. TFC was expressed as µg quercetin equivalents per mL of methanol extract and measured spectrophotometrically at 415 nm.

### 2.9. Statistical Analyses 

Statistical analyses were performed with SPSS (Version 24 for Windows Inc., Chicago, IL, USA). When the data did not follow the normal distribution, the nonparametric Kruskal–Wallis test was performed. For phytochemical analyses, normality allowed for one-way analysis of variance followed by Tukey’s test at (*p* ≤ 0.05).

## 3. Results and Discussion

### 3.1. Effect of Light Quality on Growth Parameters

The different wavelengths had variable effects on the growth of *Pistacia vera* (Table 1 and Table 2). The maximum total weight and callus weight were obtained under R, followed by BR. For shoot weight, no significant difference was found between R and BR, but both were superior to FL and B. The number of shoots and leaves per explant were not significantly affected by light quality. However, there were more and longer usable shoots under R, which, when divided, displayed a five-times-better proliferation rate than FL. Similarly to Nezami-Alanagh et al. [6], in our previous studies, we found that the proliferation rate of pistachio decreased steadily from subculture to subculture during the long-term micropropagation period [5]. However, this loss of vigor was compensated for under R in our current study. The influence of light spectrum on growth parameters has been demonstrated in several studies. Lotfi et al. [20] compared the effects of combinations of B, R, and Far-Red LED (FR) light with FL during micropropagation and rooting of *Pyrus communis,* and demonstrated the beneficial effect of R on shoot length and leaf area. The positive effect of R on stem elongation was also demonstrated in *Chrysanthemum* [28], grape [16], *Ficus benjamina* [17], and *Rehmannia glutinosa* [29]. The responses of shoots grown in the absence of B in our study mimic those associated with shade avoidance syndrome (accelerated internodal elongation and hyponasty). However, the latter is known to be triggered first by a decrease in the Red/Far-Red ratio, which is an early warning signal of upcoming competition for light, and is followed by a decrease in total light intensity and Blue light depletion [30]. Exceptionally, dim Blue light alone stimulates the elongation of internodes in some species, including *Stellaria longipes* [31] and tobacco [32]. The decrease in Blue light is recorded primarily by cryptochrome Blue light receptors, but also by reduced photosynthesis under low light conditions [33,34]. Cryptochromes belong to the Blue-light-sensitive photoreceptors, along with phototropins and members of the ZEITLUPE family [35]. They play an active role in elongation reactions [36,37]. The molecular mechanisms of the plant’s response to the lack of Blue light remain unknown [30].

Nowadays, it is generally agreed that the effects of (combinations of) wavelengths on growth parameters are species dependent. In *Pyrus communis*, for example, the maximum number of shoots was recorded under BR [20]. The ratio B:R (3:1) was beneficial for the differentiation and proliferation of Westar plants [38]. The R light spectrum promoted the induction and proliferation of protocellular bodies in *Oncidium* [39]. However, B promotes growth in *Rehmannia glutinosa* [40]. Our findings are in agreement with Huimin [38], who suggested that the effects and mechanisms associated with light quality in plants may be specific to plant species or cultivars.

### 3.2. Revealing the Effect of Light Quality on the Percentage of Hyperhydric Shoots and Severity of HH

**Figure 1:**
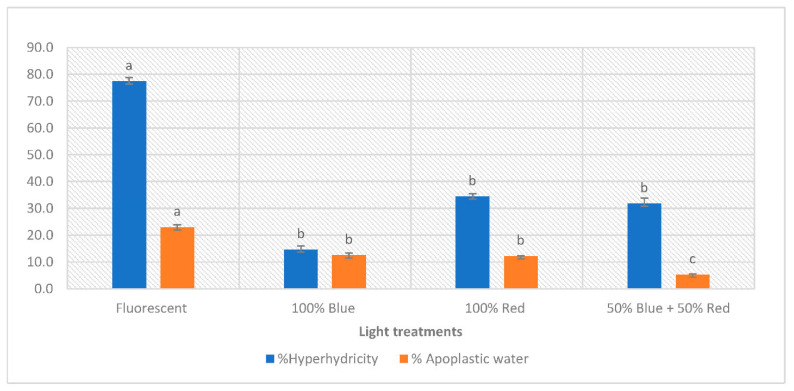
Effect of light spectrum on percentage of hyperhydric shoots and percentage of apoplastic water in leaves in *Pistacia vera* grown in vitro. Data are recorded as mean ± SE. Different letters show significant differences (*p* < 0.05), according to Kruskal–Wallis test, between light treatments.

**Figure 2:**
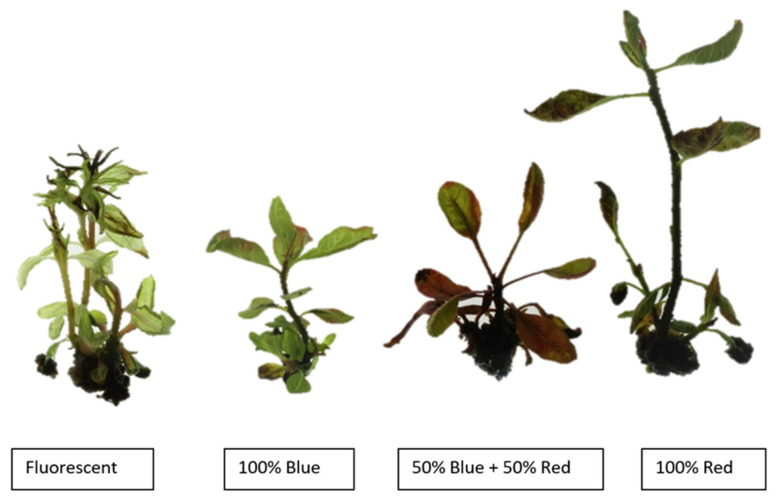
Physiological disorders harming in vitro *Pistacia vera* culture under different light qualities.

After 6 weeks, all shoots were visually scored for HH. With each light treatment, more or less severe symptoms of HH were observed, such as brittle leaves and a translucent appearance. However, their relative number depended on the wavelength (Figure 1). Under FL, shoots recorded a relatively high percentage of HH, reaching 77.4% (Figure 2). Under R, BR, and B, this percentage decreased to 34.4, 31.7, and 14.7%, respectively. HH is considered one of the most common and unfavorable physiological disorders observed during micropropagation. It is generally distinguished by specific anatomical, morphological, physiological, and metabolic changes resembling stress effects [41]. One of the main causes may be high relative humidity and gas accumulation (mostly CO_2_ and ethylene) in the environment of sealed culture vessels [42], but it may also be due to high cytokinin concentration or high water availability in the medium. Wavelength was rarely considered. Muneer et al. [43] showed that R and B can reduce HH in carnation shoots and do so by maintaining antioxidant defense mechanisms and thylakoid protein composition. Zarate-Salazar et al. [19] were also successful in reducing HH in *Lippia grata* by combining headspace aeration with BR.

Kemat et al. [21] found that HH is due to apoplastic flooding, so in order to further investigate and evaluate the severity of HH under different wavelengths, the percentage of apoplastic water volume was determined after six weeks of culture. Shoots accumulated significantly lower apoplastic water values under B, R, and BR than under FL. For R and BR, the decrease in apoplastic water content can also be explained by the severe necrotic nature of leaves developing under these lights (Figure 2). Therefore, there is a difference between the trend of the percentage of apoplastic water and the visual evaluation of HH.

The results obtained highlight the potential positive role of the various LED lamps in mitigating HH.

### 3.3. The Effect of Light Quality on LN and STN

In plants, necrosis is a common response to various environmental stresses, and is characterized by the death of cells or tissues, resulting in visible symptoms such as tissue collapse, wilting, and discoloration [44]. A variety of factors, including contamination, nutrient imbalance [45], physical damage [46] and oxidative stress [47], can cause this type of cell death during in vitro culture. Light has been also shown to play a critical role in the development of plant necrosis in vitro. High-intensity light exposure has been shown to be an inducer of necrosis in plant cells in vitro [48].

**Figure 3:**
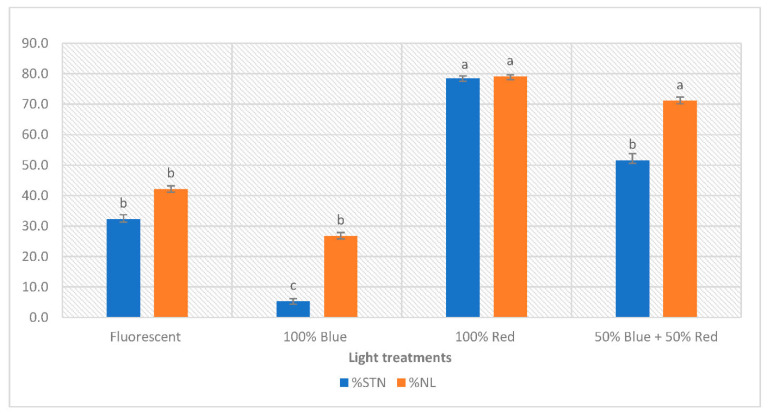
Effect of light spectrum on percentage shoot tip necrosis (STN) and leaf necrosis (LN) per usable shoot in in vitro-cultured *Pistacia vera*. Data are recorded as mean ± SE. Different letters show significant differences (*p* < 0.05), according to the Kruskal–Wallis test, between light treatments.

In our study, LN was found to be affected by light quality (Figure 3). The maximum percentage of leaves with this defect was recorded under R and BR, at 79% and 71.1%, respectively. Under B, LN was significantly lower (26.7%) than under R and BR, but the difference with FL (42.1%) was not significant. The same trend was observed for STN: shoots grown under R recorded the highest percentage of STN (78.5%), followed by RB and FL with 51.6% and 32.3%, respectively. The lowest percentage was recorded under B and was only 5.3% (Figure 3).

There is an association between LN and STN; a Chi-Square test confirmed a significant relationship (Supplementary Tables S1 and S2). All shoots with STN had at least one necrotic leaf. It is assumed that these disorders are due to calcium (Ca) deficiency [45,49]. Light is one of several factors that can affect the Ca content in the plant, and therefore might affect necrotic symptoms in the leaves and shoot tips. Olle and Bender [50] discussed the causes and control of Ca deficiency disorders in vegetables, including the light effect. They found that supplemental lighting and too-high light intensity can cause Ca deficiency in plants. In addition, light can affect plant necrosis by increasing [51] or reducing [52] the production of reactive oxygen species (ROS). ROS are highly reactive molecules that can damage cells by oxidizing cellular components such as lipids, proteins, and nucleic acids, causing necrosis [53]. Indeed, B was found to induce significantly higher DPPH radical-scavenging activity in *Pachyrhizus erosus* [52].

To our knowledge, no study has yet compared the effect of different wavelengths on necrosis defects. In our results, the effect of different wavelengths on necrosis phenomena in *Pistacia vera* has been demonstrated, but further analysis is needed to understand the exact mechanism.

### 3.4. Blue LED Light Promotes Production of Photosynthetic Pigments

Shoots grown under B yielded significantly more chlorophyll a and total chlorophyll than shoots grown under Fl and BR (Table 3). Shoots grown under R also yielded less chlorophyll a and fewer carotenoids than shoots grown under B, but the total chlorophyll content was not significantly different from that of B (Table 3). The effectiveness of B in the synthesis of chlorophyll pigments has also been reported by several studies [16,38]. Muneer et al. [54] found that B promoted the photosynthesis of lettuce plants by stimulating the expression of photosystem-related proteins. It was reported that B regulates the expression of about 6000 genes encoding photosynthetic proteins and proteins involved in cell wall biosynthesis [55]. The same authors found that inactivation of B signaling components (CRY1, CRY2, and HY5) led to delayed chlorophyll accumulation and a decrease in the expression of key genes responsible for its biosynthesis.

In our study, shoots grown under B showed the best quality compared to the other light treatments tested, with simultaneously low necrosis and HH symptoms (Figure 1, Figure 2 and Figure 3). This superior quality could be explained by an efficient photosynthesis process. However, proving the causal relationship between photosynthetic pigment production, HH, and necrosis disorders would require further molecular analysis.

### 3.5. Effect of Light Quality on the Concentration of Flavonoids, Total Phenolic Compounds, and DPPH in Pistacia vera L.

The TPC, TFC, and DPPH on *Pistacia vera* leaves exposed to different monospectral light conditions are shown in Table 4. The production of polyphenols was affected by light wavelength. The TPC doubled significantly, from 2.49 (FL) to 4.88 mg (BR) GAE/mL. R and B resulted in intermediate values that were not significantly different from FL or BR. The TFC did not differ between FL and B, but decreased when R was used, even in combination with B, by 35.50 and 10.68 µg GAE/mL, respectively. The effects on DPPH radical-scavenging activity were rather limited: leaves exposed to R showed a lower value (92.25%) compared to B and BR.

The effect of light wavelength on polyphenol production and antioxidant capacity has also been reported by several authors [40,56]. Li et al. [57] reported that the biosynthesis of secondary metabolites in plants is mediated by the light spectrum and is associated with antioxidant activities. Chung et al. [52] found that exposure to B increased TPC and TFC in *Pachyrhizus erosus* (L.) seedlings, and attributed this effect to the fact that the wavelengths of B and the UV spectrum are close to each other and thus cause similar effects during phenolic biosynthesis. Naznin et al. [58] found that the effect of light spectrum on antioxidant capacity is species dependent.

Our in vitro culture system exerts a certain level of stress on the explants, since HH, LN, and STN are always present. The reduction of HH, LN, and STN was wavelength dependent, but not related to DPPH-scavenging activity or TPC, which are considered as stress indicators. However, it is remarkable that the lower the TFC, the higher the percentage of LN.

## 4. Conclusions

In the present study, we showed that wavelength not only affects the morphology of *Pistacia vera*, but can also alter its physiology during in vitro culture. LED illumination (R, B, and BR) was found to be able to reduce the incidence of HH. While R spectacularly increased the proliferation rate, it dramatically increased STN and LN. B not only reduced HH, but also minimized STN. However, it did not cause much proliferation. Pigment content and antioxidant activities were also affected by wavelength, providing an indication of how light affects the morphology of *Pistacia vera*. Despite the benefits, the transfer of pistachio culture from FL to LED light should be carefully considered. There is no specific wavelength to solve HH, STN, and LN in combination with good proliferation. However, in practice, a sequence of R illumination to proliferate shoots followed by B illumination to avoid HH and STN could be a good compromise. To our knowledge, no study has compared this effect of different wavelengths on necrosis and HH defects in *Pistacia vera*. Therefore, further analysis, including phytochrome- and cryptochrome-dependent gene expression, are needed in order to understand the exact mechanism.

## Figures and Tables

**Table 1:** Effect of light spectrum on shoots and callus of in vitro-grown *Pistacia vera*.

Light Treatments	Shoot Weight (mg)	Callus Weight (mg)	Total Weight (mg)	Shoot Weight (%)	Callus Weight (%)
Fluorescent	196.0 ± 0.00 ^b^	192 ± 0.01 ^c^	389.6 ± 0.01 ^a^	53.4 ± 0.33 ^a^	46.6 ± 0.33 ^b^
100% Blue	166.2 ± 0.00 ^b^	179.1 ± 0.00 ^c^	345.3 ± 0.01 ^b^	49.1 ± 0.28 ^b^	50.9 ± 0.28 ^a^
100% Red	342.7 ± 0.00 ^a^	475.6 ± 0.01 ^a^	817.1 ± 0.01 ^b^	44.7 ± 0.40 ^b^	55.3 ± 0.40 ^a^
50% Blue + 50% Red	276.9 ± 0.00 ^a^	320.9 ± 0.00 ^b^	596.2 ± 0.01 ^b^	45.8 ± 0.28 ^b^	54.2 ± 0.28 ^a^

Averages ± SE followed by different letters in the same column are significantly different at *p* ≤ 0.05 according to the Kruskal–Wallis test.

**Table 2:** Effect of light spectrum on number of shoots, usable shoots, number of leaves, length of usable shoots and proliferation rate of in vitro-cultured *Pistacia vera*.

Light Treatments	Number of Shoots/Explant	Number of Usable Shoots/Explant	Length of Usable Shoots (cm)	Proliferation Rate	Total Number of Leaves
Fluorescent	3.6 ± 0.04 ^a^	0.9 ± 0.02 ^b^	1.9 ± 0.05 ^b^	1.5 ± 0.04 ^b^	10.2 ± 0.08 ^a^
100% Blue	3.8 ± 0.04 ^a^	0.7 ± 0.02 ^b^	1.3 ± 0.01 ^b^	0.8 ± 0.02 ^b^	9.7 ± 0.09 ^a^
100% Red	4.6 ±0.07 ^a^	2.6 ± 0.05 ^a^	3.7 ± 0.04 ^a^	7.3 ± 0.15 ^a^	10.4 ± 0.05 ^a^
50% Blue + 50% Red	4.4 ±0.05 ^a^	0.7 ± 0.02 ^b^	1.4 ± 0.02 ^b^	0.9 ± 0.03 ^b^	9.7 ± 0.10 ^a^

Averages ± SE followed by different letters in the same column are significantly different at *p* ≤ 0.05 according to the Kruskal–Wallis test.

**Table 3:** Effect of light spectrum on chlorophyll and carotenoid content in the leaves of *Pistacia vera* after being grown in vitro for 6 weeks.

Light Treatments	Chlorophyll ^a^ (µg /mg DW)	Chlorophyll ^b^ (µg /mg DW)	Total Chlorophyll (µg /mg DW)	Carotenoids (µg /mg DW)
Fluorescent	0.64 ± 0.02 ^c^	0.35 ± 0.01 ^a^	0.99 ± 0.03 ^b^	0.16 ± 0.01 ^ab^
100% Blue	1.14 ± 0.01 ^a^	0.54 ± 0.00 ^a^	1.67 ± 0.01 ^a^	0.26 ± 0.00 ^a^
100% Red	0.79 ± 0.01 ^bc^	0.60 ± 0.09 ^a^	1.39 ± 0.10 ^ab^	0.10 ± 0.03 ^b^
50% Blue + 50% Red	0.83 ± 0.03 ^b^	0.39 ± 0.00 ^a^	1.22 ± 0.04 ^b^	0.22 ± 0.01 ^ab^

Averages ± SE followed by different letters in the same column are significantly different at *p* ≤ 0.05 according to the Tukey test.

**Table 4:** Effect of light spectrum on total phenolic compounds (TPC), total flavonoid compounds (TFC), and DPPH pathway-capturing activity.

Light Treatments	TPC (mg GAE/mL Methanol Extract)	TFC (µg QE/mL Methanol Extract)	DPPH (%)
Fluorescent	2.49 ± 0.08 ^b^	65.54 ± 4.91 ^a^	93.74 ± 0.09 ^ab^
100% Blue	3.61 ± 0.21 ^ab^	64.15 ± 1.70 ^a^	94.20 ± 0.14 ^a^
100% Red	3.61 ± 0.31 ^ab^	35.50 ± 1.16 ^b^	92.65 ± 0.20 ^b^
50% Blue + 50% Red	4.88 ± 0.08 ^a^	10.68 ± 0.30 ^c^	94.48 ± 0.20 ^a^

Averages ± SE followed by different small letters in the same column are significantly different at *p* ≤ 0.05 according to the Tukey test.

## Data Availability

All data is contained in the manuscript and Supplementary Materials.

## Supplementary Materials

The following supporting information can be downloaded at: https://www.mdpi.com/article/10.3390/plants12071546/s1: Table S1: Result of Chi-Square Test; Table S2: Soot tip necrosis∗leaf necrosis Crosstabulation.
