# Supplementary material for: Effects of Monochromatic Light on Growth and Quality of Pistacia vera L
Source: Plants (Basel). 2023 Apr 3;12(7):1546. doi: 10.3390/plants12071546 (PMC10096592; doi:10.3390/plants12071546)
Supplement: Supplementary file 1 [file plants-12-01546-s001.zip › plants-2306925-supplementary.pdf]

Table S1. Result of Chi-Square Tests.

	Value	df	Asymptotic Significance (2-sided)	Exact Sig. (2-sided)	Exact Sig. (1-sided)
Pearson Chi-Square	36,400[a]	1	,000		
Continuity Correction[b]	34,098	1	,000		
Likelihood Ratio	47,970	1	,000		
Fisher's Exact Test				,000	,000
Linear-by-Linear Association	36,264	1	,000		
N of Valid Cases	268				

Table S2. Soot tip necrosis * leaf necrosis Crosstabulation.

			Leaf necrosis		
			absence of leaf necrosis	presence of leaf necrosis	Total
stn	No Shoot tip necrosis symptoms	Count	30	99	129
		Expected Count	14,4	114,6	129,0
		% within stn	23,3%	76,7%	100,0%
	Presence of shoot tip necrosis test symptoms	Count	0	139	139
		Expected Count	15,6	123,4	139,0
		% within stn	0,0%	100,0%	100,0%
Total		Count	30	238	268
		Expected Count	30,0	238,0	268,0
		% within stn	11,2%	88,8%	100,0%
